# GPR65 as a potential novel therapeutic target for the treatment of hepatic fibrosis

**DOI:** 10.1186/s40779-023-00507-2

**Published:** 2024-01-08

**Authors:** Leila Gobejishvili

**Affiliations:** Department of Physiology, University of Louisville School of Medicine, Louisville, KY 40202 USA

**Keywords:** Inflammation, Fibrosis, G protein coupled receptor 65, Macrophages

Hepatic fibrosis is a consequence of chronic liver disease, which can lead to cirrhosis and liver failure. There is no Food and Drugs Administration approved therapy for liver fibrosis to date; hence, identifying effective therapeutic targets is an urgent need. Hepatic macrophages play a critical role in both initiation and progression of fibrosis. While resident liver macrophages, Kupffer cells are considered more anti-inflammatory, recent view has demonstrated that monocyte-derived macrophages (MoMs) are more pro-inflammatory and pro-fibrogenic [1]. Moreover, MoMs exhibit more plasticity and undergo M1/M2 “polarization”. The research by Zhang et al. [2] identified GPR65 signaling as a novel mechanism responsible for hepatic macrophage M1 polarization during liver injury and fibrosis. Notably, the role of this receptor in modulating inflammatory responses by various cells in other tissues has been previously reported [3]. However, the role of GPR65 in liver inflammation and fibrosis has not been examined until now.

GPR65 is a member of the proton-activated G protein-coupled receptor (GPCR) family, which serves as pH sensor and is expressed in metabolically important organs, including liver [3]. GPR65 is mainly expressed in immune cells (eosinophils, CD4^+^ T cells, and macrophages). Tissue injury and inflammation is often accompanied by a local acidification and pH changes, which is sensed by various proton-activated GPCRs including GPR65. Zhang et al. [2] found that the hepatic expression of GPR65 was significantly upregulated in patients with fibrosis and in two distinct experimental mouse models of fibrosis. These observations indicate that GPR65 upregulation is not specific to a single etiology of fibrosis but rather common for fibrogenesis. The authors also showed that, within the liver cells, *Gpr65* mRNA levels were the highest in isolated liver macrophages, which increased in fibrotic liver. However, it was not clear which cells co-expressed GPR65 in the liver tissue. Relevant to its role in macrophage polarization, authors used various approaches to demonstrate that GPR65 expression was associated with pro-inflammatory M1 macrophage phenotype in vitro. The authors also addressed the role of extracellular acidification in the macrophage polarization. Specifically, they showed that acidic pH promoted the inflammatory phenotype in hepatic macrophages, partly in a GPR65-dependent manner. However, it was interesting that acidic pH did not increase GPR65, which indicates that although acidic environment in inflamed liver drives GPR65-dependent macrophage polarization, it does not affect GPR65 expression.

To show the relevance and causal relationship of GPR65 in fibrogenesis, the authors first used *Gpr65* knockout mice in their studies. Liver transcriptomic analyses demonstrated that while *Gpr65* deletion did not have a significant effect on baseline liver homeostasis, it affected various inflammatory and fibrotic pathways. Importantly, *Gpr65* deletion and pharmacological inhibition prevented the development of liver injury and fibrosis in cholestatic- and hepatotoxin-induced liver fibrosis models in mice. To further confirm that macrophage specific GPR65 was responsible for this protection, the authors performed bone marrow transplantation. Chimeric mice containing *Gpr65* knockout bone marrow cells demonstrated the protection from CCl_4_-induced hepatic fibrosis, inflammation, and injury. This protection was mediated by decreased proinflammatory cytokine expression and increased interleukin (IL)-10 levels in *Gpr65* knockout bone marrow-derived macrophages, confirming that GPR65 plays a critical role in MoM M1 polarization in vivo. Additionally, the authors showed that GPR65 signaling led to transforming growth factor-β1 (TGF-β1) production in macrophages, which identifies GPR65 as a regulator of pro-fibrogenic phenotype.

Interestingly, recent work reported that decreased extracellular pH increases TGF-β1 production in a GPR4/GPR65-dependent manner in dermal fibroblasts [4]. Results from the study by Zhang et al. [2] also showed that increased levels of *TGFβ1* mRNA in the LX2 human hepatic stellate cell (HSC) line upon their exposure to low pH, and in primary HSCs by overexpressing GPR65. Additionally, although baseline levels of GPR65 are much lower in HSCs, authors observed that the levels of HSC-GPR65 increased in fibrotic liver. These results indicate that there could be a link between acidosis, TGF-β1 production and GPR65 signaling in both macrophages and HSCs. Hence, the role of HSC-GPR65 signaling in the pathogenesis of fibrosis cannot be completely ruled out since activated HSCs also produce TGF-β1.

Perhaps the most important observation of the study was that macrophage GPR65 signaling contributed to hepatocyte apoptosis and HSC activation during fibrogenesis. The authors presented extensive data demonstrating that tumor necrosis factor-α, IL-6 and TGF-β1 released by macrophages in a GPR65 dependent manner, are key drivers of HSC activation and hepatocyte injury. This observation suggests that GPR65 plays a significant role in cell-cell communication that perpetuates hepatocyte damage and HSC activation during fibrogenesis. From a clinical perspective, this study showed that GPR65 inhibition has a therapeutic potential in attenuating key pathogenic events in fibrogenesis. It is important to point out that in these studies GPR65 inhibition/deletion not only prevented but also attenuated the development of fibrosis, which suggests that GPR65 could serve as a promising therapeutic target for liver fibrosis.

## Data Availability

Not applicable.

## Abbreviations

GPCR: G protein-coupled receptor
HSC: Hepatic stellate cell
IL: Interleukin
MoMs: Monocyte-derived macrophages
TGF-β1: Transforming growth factor-β1

## References

[1] Wang S, Friedman SL (2023). Found in translation-fibrosis in metabolic dysfunction-associated steatohepatitis (MASH). Sci Transl Med.

[2] Zhang K, Zhang MX, Meng XX, Zhu J, Wang JJ, He YF (2023). Targeting GPR65 alleviates hepatic inflammation and fibrosis by suppressing the JNK and NF-κB pathways. Mil Med Res.

[3] Imenez Silva PH, Camara NO, Wagner CA (2022). Role of proton-activated G protein-coupled receptors in pathophysiology. Am J Physiol Cell Physiol.

[4] Takano K, Kasamatsu S, Aoki M, Takahashi Y (2023). Carbon dioxide-induced decrease in extracellular pH enhances the production of extracellular matrix components by upregulating TGF-β1 expression via CREB activation in human dermal fibroblasts. Exp Dermatol.

